# Evaluation of Rubella Immunity in a Community Prenatal Clinic

**DOI:** 10.5402/2013/602130

**Published:** 2013-01-15

**Authors:** Edward C. Nwanegbo, Thor Swanson, Oluseyi Vanderpuye, Carlos F. Rios-Bedoya

**Affiliations:** ^1^Department of Family and Community Medicine, Texas Tech University and Lamb Health Care Center, Littlefield, TX 79339, USA; ^2^Siouxland Medical Educational Foundation and Siouxland Community Health Center, Sioux City, IA 51104, USA; ^3^College of Sciences and Health Professions, Albany State University, Albany, GA 31705, USA; ^4^Department of Family Medicine, Michigan State University, East Lansing, MI 48824, USA

## Abstract

Since the introduction of the Rubella vaccine in 1969, prevalence of congenital Rubella syndrome (CRS) has greatly declined in the United States. However, reports of sporadic adult cases of the disease and frequent identification of non-Rubella immune (NRI) women in prenatal units may result in outbreak of CRS in susceptible communities. Identifying populations with high rates of NRI will assist in evidence-based public health intervention that may prevent epidemic of CRS in the United States. *Method*. This is a retrospective, cross-sectional study involving chart audit of Rubella screening results of 642 women who attended a high-risk prenatal care at a northwestern Iowa clinic between January 1 and December 31, 2007. *Results*. NRI was found in 6.9% of the study population. The highest prevalence rate of 10.2% was found among adolescents. NRI was highest among Native American women at 17.3%, compared to Whites 7.3%, African Americans 5.9%, and Hispanics 4.6%. Multivariate analysis demonstrated that Native Americans were 2.5 times more likely to be NRI compared to Whites (OR 2.7; 95% CI: 1.1, 6.6). *Conclusion*. This study demonstrated higher rate of non-Rubella immunity among adolescent pregnant women and supports Rubella booster immunization for all non-pregnant teenage women. The observed high rate of NRI among Native Americans may require further studies and evaluation of Rubella vaccination programs in tribal communities.

## 1. Introduction

With the increase in international travels and multiracial communities in the United States, non-Rubella immune (NRI) pregnant women as well as nonimmune individuals are at risk of Rubella infection. A study in 1988 suggested that between 7.5 and 17.4% of pregnant women in the United States lack immunity to Rubella [1]. Similarly, a prevalence rate of 15.1% among pregnant women was reported in 1999 by investigators in Michigan [2]. In addition, a rate of 9.1% was found among women who attended prenatal care at Lejeune NC in 2004 [3]. In view of the recent report of two adult cases of Rubella infection in the United States [4], the importance of identifying populations at risk for Rubella infection cannot be over emphasized.

 Pregnant women who are NRI may be infected through exposure to asymptomatic individuals. This may result in miscarriage, fetal death, or severe anomalies in the infant with CRS [5]. Infection during the first trimester of pregnancy is associated with worst outcomes and development of multiple organ anomalies associated with CRS [6]. Some of the effects of CRS may not be apparent at birth [7] and may manifest as a chronic disease capable of producing ongoing organ damage throughout the life of an infected child [6]. 

 Although the prevalence of CRS and adult Rubella infection declined remarkably following the introduction of Rubella vaccine in 1969 in the United States [8, 9], the disease is still endemic in many developing countries. In many developing countries such as sub-Saharan Africa, there is no existing national vaccination program against Rubella, and surveillance data is limited [10, 11]. These developing countries are responsible for most of the estimated 238,000 global cases of children born annually with CRS [12]. 

 In the United States, the last major epidemic of the disease resulted in about 12.5 million cases of infection, 20 000 cases of CRS and 11,000 fetal deaths [13]. Sporadic cases of the disease still occur in the United States [14, 15] because of lack of universal immunization, primary and secondary vaccine failure and reinfections [16, 17]. Some sporadic cases in the United States have led to the association of race with Rubella infection [18]. For instance, Rangel and colleagues reported outbreak of Rubella infection in a Hispanic community in North Carolina [14]. In addition, another outbreak was reported in meat processing plants that employed many Hispanics and other foreign nationals in Kansas and Nebraska [19]. Similarly, Plotinsky and colleagues reported a case of CRS in an infant born to a Liberian refugee living in New Hampshire [19]. Furthermore, during 1997 to 1999, 81% of reported CRS cases were Hispanics and 92% of the infants were born to foreign mothers [20].

Apart from the numerous medical consequences of the disease [21–24], the economic cost of the disease is also enormous [6]. For instance, the epidemic of 1963–1965 cost an estimated 2 billion dollars [8]. Identification and protection of NRI women of child bearing age against Rubella will prevent CRS and its associated morbidity, mortality, and economic health burden.

 The objectives of this study were to evaluate the prevalence and distribution of NRI among pregnant women who attended multiracial high-risk prenatal clinic in 2007. Findings from the study may provide the rationale for targeted public health intervention that may prevent reemergence of Rubella.

## 2. Method

This is a retrospective, chart review study involving pregnant women who attended high-risk prenatal clinic in northwestern Iowa in 2007. This study was part of a routine evaluation of prenatal care in the clinic and approved by Sioux City Institutional Review Board, Iowa, United States.

The clinic serves a diverse population of low-income and multiracial groups including a large Hispanic population, non-Hispanic Whites, Native Americans, African Americans, Asians, and African immigrants living in the tri-state area of Nebraska, Iowa, and South Dakota. During their initial prenatal visit, all patients gave blood samples for routine prenatal laboratory screening. Determination of Rubella immunity was carried out as part of this screening exercise. 

### 2.1. Laboratory Test

Rubella screening was conducted by evaluating serum Rubella IgG antibody titer using Enzyme Linked Immunosorbent Assay (ELISA) (Wampole Laboratories, Princeton, NJ) as described previously [13]. Individuals with equivocal results and those with no Rubella IgG antibody were regarded as nonimmune. Records of screening results were documented in paper charts and made available to physicians. Single researcher extracted the data in Microsoft Excel (Microsoft Corp., Redmond, WA) for analysis.

### 2.2. Data Analysis

Prevalence rate in the study population was calculated by dividing the number of NRI women with the total number of women screened during the study period. 

Three age groupings were used in the analysis of data. These include less than 20 years of age, or adolescent, 20–29 years of age and 30 or more years of age. NRI prevalence rates in these age groups were calculated by dividing the number of NRI in the age group by the total number of women screened in that age group. Furthermore, we evaluated the prevalence rates in four major study populations, namely, Hispanics, Whites, Native Americans, and African Americans.

Data was analyzed using Sigma Stat software (Jandel Scientific, San Rafael, CA). Odds ratio (OR) was established at 95% confidence interval. Statistical significance was established using Chi square or Fisher's exact test. To control for possible differences in age distribution across race/ethnic groups, a logistic regression analysis was conducted. Presence or absence of Rubella immunity was the response variable. Race/ethnic group was the primary explanatory variable, and whites were selected as the reference category for comparison purposes. Age was added to the unadjusted logistic model to determine the relationship between race/ethnicity and the presence or absence of Rubella immunity while holding constant the influence of age.

## 3. Results

### 3.1. Study Population

A total of 642 women consented for prenatal screening in 2007. Results were available for 641 or 99.8%. Patients who identified themselves as Hispanics constituted 54.2% of the study population. Non-Hispanic population comprised of Whites or Caucasians (180), African Americans (34), Native Americans (52), Asians (21), and others (7). The Asians, Biracial (3), and Africans from Somalia (4) were in the group classified as “others” and will not be treated as a racial group. Women aged 20–29 years of age constituted the highest number of subjects and accounted for 57.9% of the study population. Adolescents and women 30 or more years of age accounted for 18.4% and 23.7% of the study population, respectively. 

### 3.2. Prevalence Rates in the Study Population

**Figure 1:**
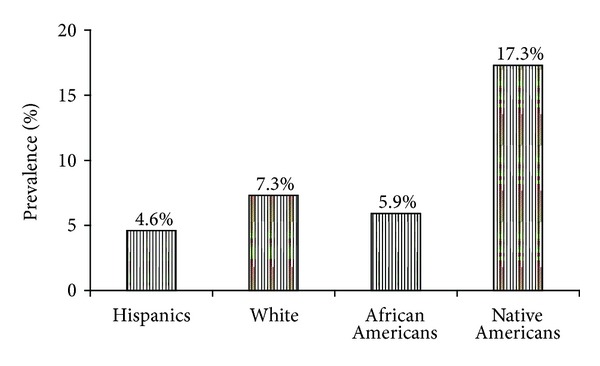
Prevalence rate of non-Rubella immunity (NRI) in the study population. Native Americans demonstrated the highest rate at 17.3% (Hisp: Hispanics; Afr Am: African Americans; Nat Am: Native Americans).

A total of 44 women were NRI in the study population. Prevalence rate in the study population was 6.9%. The prevalence rate among Native Americans was highest at 17.3%, compared to Whites 7.3%, Blacks 5.9%, and Hispanics 4.6% (see Figure 1).

### 3.3. Age Group Rates

**Figure 2:**
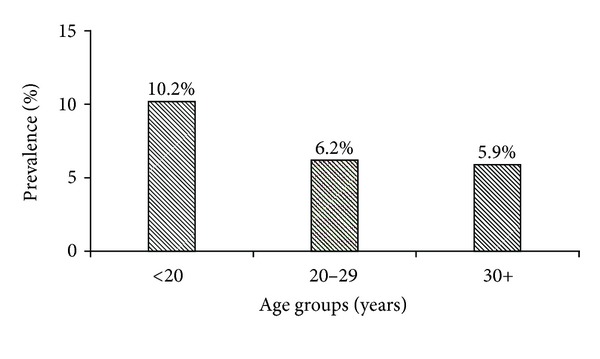
Prevalence rate of non-Rubella immunity in the three age groups. More than 10% of adolescents were non-Rubella immune compared to 6.2 and 5.9 in age groups 20–29 and 30+, respectively.

**Figure 3:**
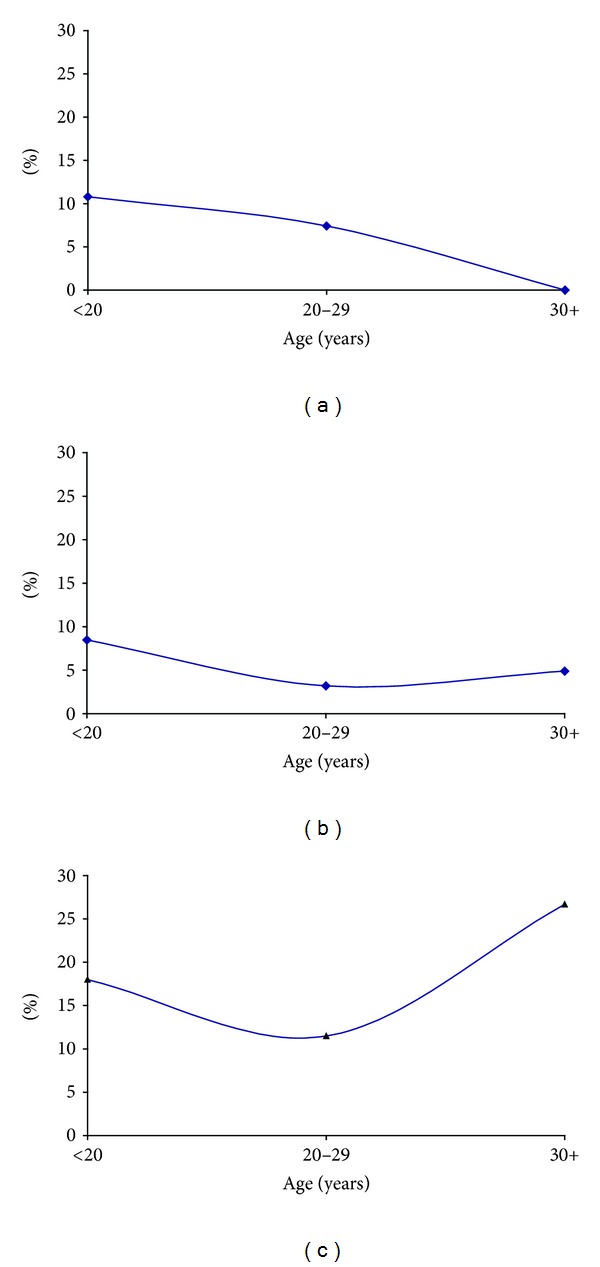
Distribution of non-Rubella immunity among Whites (a), Hispanics (b) and Native Americans (c). The study population was divided into 3 age groups, namely, under 20 years of age or adolescents, 20–29 years of age and 30 or more years of age. Prevalence rate of NRI was calculated in each age group. Distribution of NRI among Whites (a), Hispanics (b), and Native Americans (c). NRI persisted in all age groups among Native Americans at a higher rate when compared to Hispanics and Whites.

In the study population, prevalence rate among adolescents was 10.2% compared to 6.2% and 5.9% among women aged 20–29 and 30 or more years of age, respectively (see Figure 2). Analysis of the trend in three major racial groups demonstrated a high rate among Native American teenagers at 18.2% compared to Whites and Hispanics at 10.8% and 8.5%, respectively. In women aged 20–29 years of age, the rate among Native Americans was 11.5% compared to 7.4% and 3.2% of Whites and Hispanics, respectively. Among older women (30 years or more), the prevalence rate among Native Americans was 26.7% compared to 0% and 4.9% of Whites and Hispanics, respectively. The overall trend in NRI in the three main racial groups demonstrated persistence of NRI at a higher rate among Native Americans compared to Whites and Hispanics (see Figure 3).

 Crude and adjusted multivariate analysis demonstrated that Native Americans were more than two and a half times more likely not to have Rubella immunity than Whites (OR 2.7; 95% CI: 1.1, 6.6). Once we control for suspected differences in age distribution, the association remained (OR 2.7; 95% CI: 1.1, 6.7).

## 4. Discussion

This study evaluated NRI among women attending high-risk prenatal care in Iowa, United States. The NRI prevalence rate of 6.9% was demonstrated in the study population. Native American women demonstrated the highest rate at 17.2%. This race also demonstrated high prevalence rates across all three age groups. 

 The prevalence rate of NRI demonstrated in this study appeared lower compared to previous studies in 1988 (7.5%–17.4%) [1], 1999 (15.4%) [2], and 2004 (9.1%) [3].

One of the important findings of this study is the presence of comparatively higher NRI among adolescents in the study population (see Figure 2). This finding supports Rubella booster vaccination for nonpregnant women such as high school female teenagers. This may provide lifelong immunity for women during their reproductive years and prevent CRS. Secondly, booster vaccination may also prevent undiagnosed cases of Rubella that resulted in congenital anomalies [5] and first trimester loss of pregnancy. Since there is no effective treatment of infection during pregnancy that will prevent CRS, protecting women before pregnancy is therefore an attractive public health intervention against CRS. 

 Another important implication of this study is the finding of significant prevalence of NRI across all age groups among the Native American women. Compared to Whites, Native Americans are more likely to be non-immune to Rubella. This finding calls for further studies and evaluation of MMR vaccination programs existing in Native American communities. Replication of the findings in this study may warrant public health interventions aimed at protecting these communities from Rubella epidemic.

Despite the important findings of this study, it should be seen as a pilot study because of the imbalance in the study population. However, the study may provide the basis for evaluation of longevity of immunity derived from Rubella vaccine currently administered to children in the United States as part of MMR vaccine. Similarly, this paper may provide the rationale for further studies in Native American communities targeted at elucidating factors that foster decreased immunity to Rubella.
